# Stimulus-specific regulation of visual oddball differentiation in posterior parietal cortex

**DOI:** 10.1038/s41598-020-70448-6

**Published:** 2020-08-18

**Authors:** Zhe Charles Zhou, Wei Angel Huang, Yiyi Yu, Ehsan Negahbani, Iain M. Stitt, Morgan L. Alexander, Jordan P. Hamm, Hiroyuki K. Kato, Flavio Fröhlich

**Affiliations:** 1 Department of Psychiatry, University of North Carolina at Chapel Hill, 116 Manning Drive, 6018A, Chapel Hill, NC 27599 USA; 2 Department of Cell Biology and Physiology, University of North Carolina at Chapel Hill, Chapel Hill, NC 27599 USA; 3 Department of Biomedical Engineering, University of North Carolina at Chapel Hill, Chapel Hill, NC 27599 USA; 4 Department of Neurology, University of North Carolina at Chapel Hill, Chapel Hill, NC 27599 USA; 5 Neuroscience Center, University of North Carolina at Chapel Hill, Chapel Hill, NC 27599 USA; 6 Neurobiology Curriculum, University of North Carolina at Chapel Hill, Chapel Hill, NC 27599 USA; 7 Carolina Center for Neurostimulation, University of North Carolina, Chapel Hill, NC 27599 USA; 8 Neuroscience Institute, Georgia State University, Atlanta, GA 30302 USA; 9 Department of Biomedical Sciences, University of California at Santa Barbara, Los Angeles, CA 90048 USA; 10 Carolina Institute for Developmental Disabilities, University of North Carolina at Chapel Hill, Chapel Hill, NC 27599 USA

**Keywords:** Neuroscience, Sensory processing

## Abstract

The frequency at which a stimulus is presented determines how it is interpreted. For example, a repeated image may be of less interest than an image that violates the prior sequence. This process involves integration of sensory information and internal representations of stimulus history, functions carried out in higher-order sensory areas such as the posterior parietal cortex (PPC). Thus far, there are few detailed reports investigating the single-neuron mechanisms for processing of stimulus presentation frequency in PPC. To address this gap in knowledge, we recorded PPC activity using 2-photon calcium imaging and electrophysiology during a visual oddball paradigm. Calcium imaging results reveal differentiation at the level of single neurons for frequent versus rare conditions which varied depending on whether the stimulus was preferred or non-preferred by the recorded neural population. Such differentiation of oddball conditions was mediated primarily by stimulus-independent adaptation in the frequent condition.

## Introduction

Behavioral flexibility requires the brain to generate distinct patterns of neural responses when a stimulus is observed frequently versus rarely. For example, a wild deer that encounters a car on a daily basis may perceive rare, intermittent encounters differently. The latter case is much more surprising since the stimulus (driving car) possesses substantially different features from the usual experienced environment. Accordingly, there must be circuitry in the brain that is able to differentiate such scenarios.

The oddball paradigm is a classic sensory stimulus assay used to evaluate neural differentiation of stimulus presentation frequency^1,2^. In this paradigm, the neural response to a rare, deviant stimulus is compared to the response to a frequently-presented standard stimulus; differentiation of the two conditions is quantified as the difference in responses between the standard and deviant (called mismatch negativity). Specifically, the mismatch negativity (MMN) is a neurophysiological difference waveform peaking in the 150–250 ms window after stimulus onset. The MMN refers to the difference in evoked response to the standard and deviant stimulus. Importantly, the MMN is distinct from the N100 (occurring between 50 and 150 ms post stimulus onset) and the P300 (occurring around 300–400 ms) components with respect to function and timing relative to stimulus onset^3,4^. The MMN has been studied comprehensively in humans using EEG, MEG, and fMRI^5–10^, and to a lesser extent, in animal models with local field potential (LFP) and/or single units^11–16^. Yet, few studies have extensively probed how individual neurons exhibit responses to oddball conditions as a function of stimulus properties. Stimulus preference and tuning in single neurons may influence MMN responses, highlighting the need for neural recordings at higher spatial granularity using methods such as 2-photon calcium imaging.

It has been postulated that the MMN is a precursor to sensory discrimination^17,18^. Within this framework, sensory areas such as visual or parietal cortex may show differing levels of activity to the surprising deviant stimulus versus the frequently presented standard stimulus, which both hold behavioral relevance to the animal. However, whether the MMN is dominated by adaptation to the standard condition or an enhanced response to the deviant condition remains a matter of debate^17,19^. Further, heterogeneity in stimulus tuning across neurons in sensory cortices may affect oddball responses where neurons that show stronger baseline responses to a particular stimulus (e.g., motion in one direction versus the other) exhibit larger magnitude MMN compared to a stimulus with weaker baseline responses. Accordingly, neurons encoding different stimulus features effectively compete to relay behaviorally-relevant information to downstream areas.

Posterior parietal cortex (PPC) is well positioned in the visual circuit to transform basic sensory tuning to signals reflecting the frequency of stimulus presentation due to its functional involvement in stimulus motion processing and history-dependent evidence accumulation^20–22^. Importantly, PPC specializes in differentiating stimulus motion directions: upstream visual brain regions from PPC encode orientation tuning, whereas sensory responses in PPC show stronger responses to one direction over the opposite. Further, single neurons in PPC can exhibit unique tuning to different directions^22,23^. Given this direction tuning hallmark of PPC function, MMN responses may differ as a function of a neuron’s direction tuning in this brain area.

To understand how PPC neurons modulate their activity in response to stimuli presented in frequent and rare conditions, we recorded extracellular electrophysiology and performed 2-photon calcium imaging in PPC of head-fixed ferrets during a drifting grating visual oddball paradigm. Our results indicate that PPC LFP and single neuron calcium imaging activity exhibit stimulus-specific modulation across oddball conditions. Spectral decomposition of the LFP revealed MMN in the gamma frequency band for the preferred stimulus only. We leveraged the strength of 2-photon calcium imaging by comparing oddball activity for each spatially-resolved neuron and found that mismatch responses (MMR, equivalent to MMN but for calcium imaging data) scaled in magnitude with neuronal tuning to the population-preferred stimulus. We further dissected the components of the MMR and discovered that it was dominated primarily by stimulus-specific adaptation.

## Materials and methods

### Subjects

A total of six adult (16–19 weeks old) female ferrets (*Mustela putorius furo*, weighing 0.7–1 kg, group housed in a 12 h light/12 h dark cycle; spayed, Marshall BioResources, North Rose, NY) were included in this study. Three animals were included in the electrophysiological experiment (across these animals, we acquired 15 sessions) and four were included in the 2-photon calcium imaging experiment (across these animals, we acquired 21 sessions). All animal procedures were performed in compliance with the National Institutes of Health guide for the care and use of laboratory animals (NIH Publications 8th edition, 2011), and the United States Department of Agriculture (USDA Animal Welfare).

### Electrophysiology surgery

Each animal was initially anesthetized with a ketamine/xylazine cocktail (30 mg/kg of ketamine, 1–2 mg/kg of xylazine, intramuscular) and was checked for toe-pinch response for a stable, deep plane of anesthesia before continuing with intubation with an endotracheal tube for subsequent artificial ventilation and anesthetic maintenance (0.5–2% isoflurane in 100% oxygen). The animal was then positioned into a stereotaxic frame to allow for precise measurement of target brain regions and to provide stability of the head throughout the surgical procedure. All following surgical procedures were performed under asceptic conditions. After applying alcohol and betadine swabs to the scalp, the skin and muscle overlying the skull were cut and resected. A custom-machined headpost was secured to the area of the skull above the right parietal cortex and dental cement (C&B metabond, Parkell, Edgewood, NY) was applied to the base of the headpost. Craniotomy for the left parietal cortex was drilled (13.5 mm anterior to the caudal crest landmark, 3–4 mm lateral from midline) and dura mater and pia mater were resected. Sixteen channel electrode arrays (200 μm distance between each electrode; local reference electrode 500 μm shorter than recording electrodes; Innovative Neurophysiology, Durham, NC) were then lowered into cortical tissue at the depth of around 600 μm. Dental acrylic (Lang Dental, Wheeling, IL) was applied to secure electrodes and seal the craniotomy. Skin, connective tissue, and muscle were then sutured together; triple antibiotic ointment was applied to the wound margin and animals were administered pain relief medication (meloxicam, 0.2 mg/kg). Post-operative care included pain medication (meloxicam, 0.2 mg/kg) and infection control (12.5–13 mg/kg, clavamox, Zoetis, Parsippany, NJ) in addition to headcap cleaning.

### Calcium imaging surgery

Animals in the calcium imaging group (n = 4) underwent two surgeries: virus injection and cranial window implantation. Surgery procedures for the virus injection were similar to that of the electrophysiology group with the following differences: a smaller craniotomy above PPC was drilled (~ 2–3 mm in diameter). After the dura was resected, a mix of 800 nL of AAV9.CaMKII.GCaMP6f.WPRE.SV40 (viral titer = 7.55e12, UNC Viral Vector Core) and 200 nL of 1% fast green in saline was injected into cortical tissue at a depth of about 200–400 μm below cortex using a glass pipette injector system (Nanoject, Drummond Scientific, Broomall, PA). The virus/fast green solution was injected in 31 bouts of 32.2 nL at 46 nL/s with about 15 s of wait time between each bout. The tip of the glass pipette was sharp enough to penetrate through the intact pia mater with minimal damage. The craniotomy was then sealed using kwik-kast (World Precision Instruments, Sarasota, FL); then muscle, tissue, and skin were sutured back together. Cranial window surgeries were closely modelled based on a published methods paper^24^.

After around 3–5 weeks of virus expression time, the same animals underwent the cranial window surgery. The goal of the cranial window surgery was to make a larger craniotomy around the virus expression site and implant a custom coverslip holder (a machined metal ring-like structure that holds and secures a coverslip) and a glass coverslip above cortical tissue to allow for optical access. After animals were prepared for asceptic surgery and tissue overlying the skull was resected, an 8 mm circular area around the virus injection craniotomy was shaved down; this ultimately allows the coverslip structure to be as close to the brain as possible. The coverslip holder was then secured to the skull using C&B metabond and the exposed skull in the middle of the ring was then drilled out. Dura was then resected and a coverslip assembly (an 8 mm, 200 μm thick circular coverslip optical glued to a 5 mm, 1.4 mm thick cylindric glass piece; Swiftglass, Elmira, NY) was lowered into the coverslip holder. Importantly, the coverslip pressed firmly on the brain which prevents dura regrowth under the coverslip and stabilizes the brain during imaging. A retaining ring (McMaster-Carr, Atlanta, GA) was secured above the glass coverslip and superglue was applied to seal the structure. Recording sessions were made about 8–12 weeks post virus injection surgery. It is possible that such a period of time between virus injection and data acquisition may result in overexpression of GCaMP6f; however upon inspection of mean fluorescence images (Supplementary Fig. 1), we found at most 2–3 cells out of each animal’s FOV to exhibit nuclear filling.

### Oddball stimulation paradigm

Prior to surgery, animals were habituated to a custom, 3d-printed fixation setup that included a body holding tube and a head-fixation post (Thorlabs posts, Thorlabs, Newton, NJ; and custom-machined head-post clamp). After habituation and electrode/window implantation surgery, visual oddball stimulation and recordings commenced. Visual stimulation was displayed via presentation software (Neurobehavioral Systems, Berkeley, CA) on a computer monitor (120 frame rate) positioned 40 cm away from the animal. The oddball stimulation paradigm was designed as follows: stimuli consisted of grayscale full-field sine wave drifting gratings with a spatial frequency of 0.2 cycles per degree and drifting at 4 Hz. On the first day of neurophysiological recordings, animals were administered a session consisting of 10 drifting gratings of different motion directions (0°, 36°, 72°, 108°, 144°, 180°, 216°, 252°, 288°, 324°) presented at equiprobability to assess which stimuli elicited the most activity from the population. Typically the stimulus evoking the largest amplitude response during stimulus presentation (0–400 ms) and opposing direction stimulus were chosen for the following sessions. The following oddball paradigm sessions consisted of three randomized blocks of 300 trials where each trial consisted of stimulus presentation for 400 ms and an equi-luminance gray screen inter-trial interval (ITI) following for 1,000–1,500 ms. Trial order was randomized for each block. In one block (oddball A), the two best responsive stimuli from the first session were chosen to be presented in either the standard (90% of trials) or deviant (10% of trials) condition (ie. stimulus A was the deviant and B was the standard). In another block (oddball B), the same two stimuli were used but the conditions were switched (ie. stimulus B was the deviant and A was the standard). The reason for presenting both of these blocks is to be able to compare the same stimulus presented in different conditions, thus removing the confounding factor of stimulus-preference when examining neural activity. A third block’s (many-standards control block) stimuli consisted of 10 equiprobable drifting gratings of different motion directions (same structure and stimuli as the initial tuning curve session); stimuli presented in this third condition served as controls that do not elicit adaptation effects of the standard condition or surprise effects like the deviant condition. A series of random salt-and-pepper images (white noise checkerboard—10 × 6 squares, 33.3 ms presentation with 1,000–1,500 ms ITI gray screen) were presented in between the oddball paradigm blocks to reset the visual system and minimize adaptation (method adapted from a previous publication^15^).

### Electrophysiology sessions

Animals were first placed into a custom 3d-printed tube and head-fixed using a custom-machined head-post clamp. Broadband signals (1 Hz hardware highpass cutoff) from PPC were acquired at a 30 kHz sampling rate and digitized using an electrophysiology data acquisition system (Intan Technologies, Los Angeles, CA). The electrophysiology system also recorded TTL pulses sent from a separate computer running the visual stimulation via a data acquisition system (DAQ, Texas Instruments, Dallas, TX). All recording sessions were performed in a dark room save the computer monitor that displayed visual stimuli.

### Two-photon imaging

Recording of optically-resolved population GCaMP activity was performed using a resonant scanner, 2-photon microscope (Neurolabware, Los Angeles, CA), 16 × water-immersion objective (Nikon, 0.8 NA, 3 mm working distance), and the Scanbox acquisition software (Scanbox, Los Angeles, CA). Two-photon excitation of GCaMP fluorophores was delivered through the microscope using a Mai-tai Tai Sapphire laser (Spectra Physics, Santa Clara, CA) at 940 nm, and light emission was collected using a GaAsP photo-multiplier tube. Data were acquired at the frame rate of 15.49 fps. At the beginning of each session, the objective was lowered to a focal plane of 150–200 μm below the cortical surface, around layer II/III of the ferret cortex^25^. Data were typically acquired at 1.4 × digital zoom, resulting in a field of view length and width of about 800 um.

### Electrophysiology analysis

All analyses were performed in Matlab (Mathworks, Natick, MA). All broadband electrophysiological data were preprocessed in three different ways for event-related potential (ERP), spectral decomposition, and spiking analysis. For ERP analysis, broadband data were first low-pass filtered at 30 Hz using a 4th order, phase-preserving butterworth filter then downsampled to 300 Hz. For spectral analysis, broadband data were first low-pass filtered at 300 Hz using a 4th order, phase-preserving butterworth filter then downsampled to 300 Hz. For spiking analysis, broadband data were first band-pass filtered between 300 and 5,000 Hz using a 4th order butterworth filter. Spike events were detected in each channel whenever the signal crossed minus 4 standard deviations.

ERPs were calculated by extracting data in a window around each trial onset, then baseline corrected by subtracting the mean activity from − 100 to 0 ms relative to stimulus onset. Note that all times reported are relative to stimulus onset. Data were first averaged across trials for each oddball condition and stimulus, then averaged across recording channels and sessions. Statistical comparisons and standard error of the mean (SEM) were calculated across sessions. Condition contrasts (MMN: deviant-standard, DD: deviant-control) were calculated at the level of channels after averaging over trials.

For spectral analysis, we wanted to examine changes in ongoing oscillations that are not phase-locked to stimulus presentation (induced oscillations). Accordingly, the average ERP across trials was calculated and subtracted from each trial before subsequent analysis. The analytic signal for each frequency was then calculated by convolving a family of Morlet wavelets with the LFP from 1 to 50 Hz in 0.5 Hz increments. Power was then calculated as the absolute value of the analytic signal squared. Data for each trial were then extracted and averaged for each condition and stimulus. Finally, data were averaged across recording channels and sessions. Stats and standard error of the mean (SEM) were calculated across sessions.

### Calcium imaging analysis

Calcium imaging video data were first preprocessed using the Suite2p toolbox^26^. Using this toolbox, images were aligned using non-rigid motion correction and subject to singular value decomposition (SVD) across time to reduce dimensionality and reduce subsequent computation time. Pixels in the image were then clustered into regions-of-interest (ROIs) based on optimization of their spatial footprint and activity time-series using a model loosely based on expectation–maximization (EM). Using these ROIs, fluorescence activity was calculated by first taking the average time-series across pixels within the ROI; then neuropil background activity was estimated by defining a ring area around the ROI (inner diameter = 3 pixels away from ROI border, outer diameter = 5 pixels away), calculating principal component analysis (PCA) across pixels in the ring, and taking the first principal component’s (PC) activity; finally neuropil-corrected time-courses were calculated by subtracting the neuropil 1st PC activity from that of the ROI. Change in fluorescence was defined as $\Delta F/F\left(t\right)=(F\left(t\right)-{F}_{avg})/{F}_{avg}$ where $F\left(t\right)$ is the fluorescence at a given time t and ${F}_{avg}$ is the average fluorescence across the whole session.

ROIs that exhibited stimulus-evoked activity (300–1,500 ms) 1.5 standard deviations above baseline (− 200 to 0 ms) mean activity for at least 400 consecutive milliseconds for at least 25% of trials, and 1.5 standard deviations above baseline mean activity for at least 400 consecutive milliseconds in the trial average were identified as visually responsive and were included for further analysis (n = 73). Activity for each trial was then extracted and averaged for each condition and stimulus. All of the calcium imaging preprocessing steps thus far are summarized in Fig. 3D. Time-courses were baseline corrected by subtracting the mean activity from − 200 to 0 s relative to stimulus onset. Data were averaged across recording ROIs and sessions. Statistics and standard error of the mean (SEM) were calculated across ROIs. Due to the slow decay kinetics of the GCaMP6f fluorescence indicator, it is difficult to interpret when certain processes are occurring during periods of neural activity. Accordingly, we chose the period of time that calcium responses largely decayed back to baseline (from 300 to 1,500 ms relative to stimulus onset) when performing statistics or averaging across the epoch of time related to stimulus response. The comparison between standard and deviant conditions for the calcium imaging data was called mismatch response (MMR) due to the GCaMP protein exhibiting primarily positive increases in activity and its slow decay kinetics. Condition contrasts (MMR: deviant-standard, DD: deviant-control) were calculated at the level of ROIs after averaging over trials.

A concern arises when comparing activity between conditions for a certain stimulus: observed differences could arise and be confounded by block-to-block differences in baseline fluorescence. To account for this concern, we baseline corrected all time-courses on a trial level. Further, when calculating the condition contrasts, we performed a trial condition shuffle control. This first involved computing trial-averaged activity with trial conditions shuffled within each block (effectively preserving block-to-block differences in activity, while eliminating condition-dependent differences). The condition contrast was then computed (MMR or DD). Finally, trial-averaged shuffle data were subtracted from trial condition-intact data on a ROI-by-ROI basis.

To quantify an ROI’s stimulus preference, a modulation index was calculated: $\frac{{S}_{pref}-{S}_{Opp}}{{S}_{pref}+{S}_{Opp}}$ where ${S}_{Pref}$ and ${S}_{Opp}$ corresponded to the trial-averaged response during the 300–1,500 ms epoch to the preferred stimulus and opponent stimulus, respectively. This modulation index was calculated for both the MMR and control activity for each ROI.

All figures were prepared using Adobe Illustrator CS6 (version 16.0.3; San Jose, CA).

### Ethical approval

All animal procedures were performed in compliance with the National Institutes of Health guide for the care and use of laboratory animals (NIH Publications 8th edition, 2011), and approved by the Institutional Animal Care and Use Committee of the University of North Carolina at Chapel Hill and the United States Department of Agriculture (USDA Animal Welfare).

## Results

**Figure 1:**
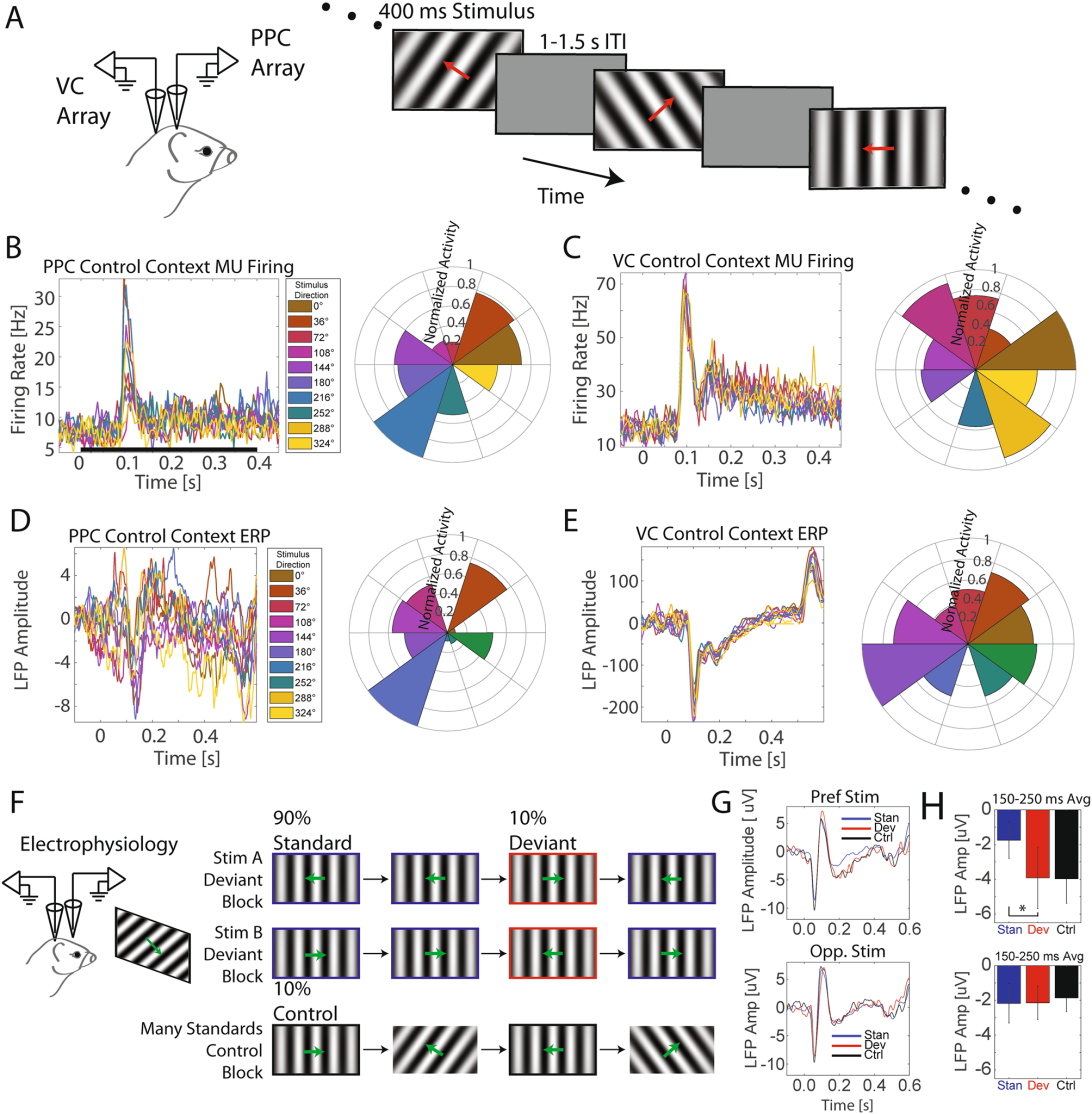
Posterior parietal cortex (PPC) multi-unit firing activity and event-related potentials (ERPs) exhibit tuning to drifting gratings in opposing directions and stimulus-specific mismatch negativity (MMN). (**A**) To assess population tuning, head-fixed ferrets were presented a series of drifting gratings during simultaneous electrophysiology in visual cortex (VC) and PPC. (**B**) Trial-averaged event-related multi-unit firing for each drifting grating direction (indicated by color) for a representative animal. Black bar represents the duration of stimulus presentation. Right: Polar histogram of normalized time-averaged (300–1,500 ms) responses as a function of drifting grating direction. Note the two opposing directions that elicit the largest responses. This indicates the presence of orientation tuning. In addition, one of the two directions exhibited a stronger response than the opposing direction, confirming the presence of direction tuning (differential response to two stimuli of identical orientation but opposing directions). The direction with the strongest response is referred to as the preferred stimulus, the opposite direction is referred to as the opponent stimulus. (**C**) Event-related firing responses in VC as a function of stimulus direction for the same animal as in (**B**). (**D**) Same format and data from same animal as in (**B**) but with PPC event-related local field potential (LFP) response. Polar histogram on the right indicates peak magnitude of the LFP during the 150–250 ms MMR window after stimulus presentation. (**E**) Same format and data from same animal as in (**D**) but ERP responses from VC. (**F**) Electrophysiology experiment in PPC during visual oddball paradigm. Each session consisted of three randomized blocks of 300 randomized trials. Drifting grating stimuli of opposing motion directions were presented for 400 ms with an inter-trial interval of 1,000–1,500 ms. The three blocks allowed for comparing the responses to the same stimulus across the standard (frequent presentation: 90% of trials), deviant (rare presentation: 10% of trials), and control (rare, but without a history of a repeated stimulus: 10% of trials) contexts. (**G**) Session-averaged (n = 15) ERPs as a function of stimulus (top and bottom panels) and context (colored traces) for PPC. PPC exhibited significant mismatch negativity (MMN) (*p < 0.05) during the classical 150–250 ms MMN epoch. Time zero represents stimulus onset for all following plots. (**H**) Time-averaged (150–250 ms) ERP responses as a function of context (bar color) and stimulus (top: preferred, bottom: opponent stimulus). Error bar represents SEM. *p < 0.05.

To dissect how processing of oddball conditions occurs as a function of stimulus preference, we first delineated stimulus tuning by presenting a series of randomized drifting gratings to head-fixed ferrets during extracellular electrophysiology (Fig. 1A, n = 3 animals) recording sessions. We found that ferret posterior parietal cortex (PPC) firing activity and local field potentials (LFP) primarily showed strong responses to two opposing directions, similar to reports in other animal species (Fig. 1B,D)^20,22^. The stimulus direction that elicited the largest normalized amplitude was deemed the preferred stimulus and the stimulus drifting in the opposite direction, which typically showed the second largest response, was deemed the opponent stimulus. PPC tuning was roughly maintained across sessions (Supplementary Fig. 2). The magnitude of the non-preferred stimulus increased over the sessions. This could be explained by plasticity of PPC tuning over the course of repeated presentation of stimuli; however testing this hypothesis was outside the scope of the current study. We did not however observe such stereotyped tuning in two specific opposing directions in visual cortex (VC) firing or LFP (Fig. 1C,E). This tuning to opposing directions that elicit the strongest responses confirms the presence of direction tuning in ferret PPC (differential response to two stimuli in identical orientation but opposing directions)^27^.

We next sought to examine electrophysiological responses to the preferred and the opponent stimulus when presented in the standard, deviant, and control conditions of the oddball paradigm (Fig. 1F). Importantly, this version of the oddball paradigm is rapidly becoming the new standard in the field since it allows for the comparison of neuronal responses across conditions for each stimulus^1,2,12^. We found a significantly larger magnitude negative deflection in the event-related potential (150–250 ms MMN epoch, Fig. 1G) for the population-preferred stimulus presented in the deviant conditions compared to that of in the standard conditions (Fig. 1H, preferred stimulus: paired *t* test; t(14) = 2.16, *p* = 0.048). Such conditions differentiation was not found for the opponent stimulus (paired *t* test; t(14) = 0.252, *p* = 0.805). MMN was not found to be significant for either stimuli in the VC ERPs (Supplementary Fig. 3). Although the difference between standard and deviant responses for Stimulus A is trending (t(6) = 2.36, *p* = 0.056), these results are difficult to interpret since the oddball stimuli were chosen based on PPC preference. Furthermore, we found no differences in spiking magnitude across oddball conditions (Supplementary Fig. 4). It is possible, at least for PPC, that the MMN is a specialized signal present in select cells and is not immediately apparent in the general neuron-averaged multi-unit firing rate.

**Figure 2:**
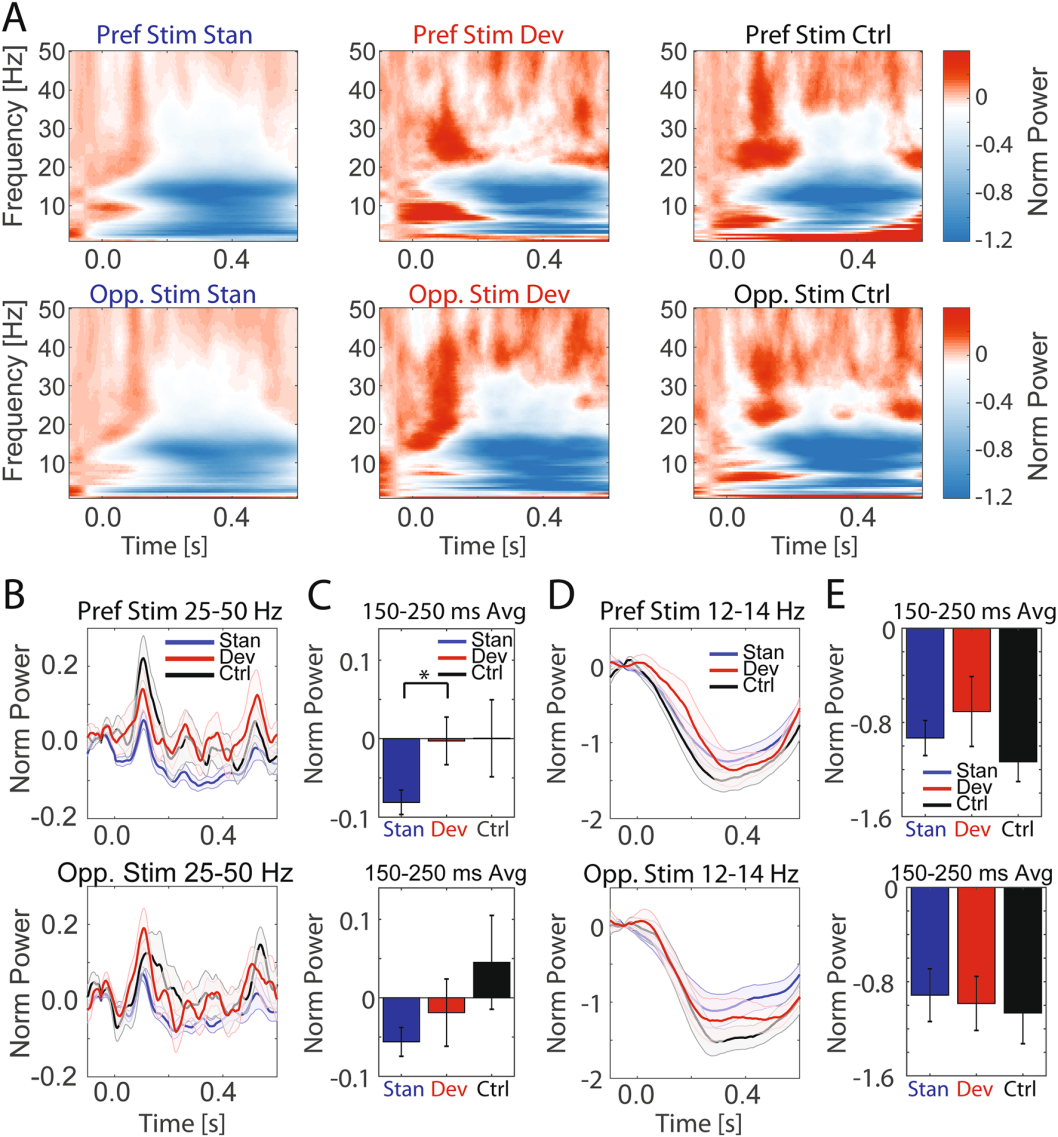
Stimulus-specific MMN is observed in the gamma frequency band. (**A**) Power spectrograms for event-related PPC activity as a function of condition (standard, deviant, control in left, middle, and right columns respectively) and stimuli (top row: preferred, bottom row: opponent stimulus). Color represents power normalized to a baseline pre-stimulus epoch (− 100 to 0 ms relative to stimulus onset) on a trial basis. Data are averaged across sessions (n = 15). (**B**,**C**) Frequency-averaged gamma frequency band (25–50 Hz) power fluctuations in PPC as a function of condition (traces) and stimulus (top row: preferred, bottom row: opponent stimulus). (**C**) Time-averaged quantification of the data in (**B**). For the preferred stimulus, PPC exhibited significantly higher gamma power for the deviant compared to the standard condition during the 150–250 ms MMN epoch (paired *t *test, *p < 0.05). Shaded error bars: SEM computed across sessions. (**D**,**E**) Frequency-averaged alpha frequency band (12–14 Hz, centered at the individual alpha peak frequency of the animal) power fluctuations in PPC as a function of condition (traces) and stimulus (top row: preferred, bottom row: opponent stimulus). (**D**) Time-averaged quantification of the data in (**E**). No differences were found between conditions in the MMN epoch for either stimulus. Shaded error bars: SEM computed across sessions.

We noticed differences in rhythmic content of the responses across conditions and followed up with spectral decomposition analysis of the LFP (Fig. 2A, event-related spectral perturbation). When we analyzed the 150–250 ms MMN epoch, we found a significant difference in the 25–50 Hz gamma frequency band power between the preferred stimulus standard and deviant conditions, where the deviant condition exhibited higher power (Fig. 2B,C, paired *t* test; t(14) = 2.25, *p* = 0.041), but not for the opponent stimulus (paired *t* test; t(14) = 0.860, *p* = 0.404). The alpha frequency band is another functional oscillatory frequency range related to visual processing, attention, and arousal^28–33^. To examine if oddball condition differentiation was specific to the gamma frequency band or a general feature across multiple frequency bands, we calculated alpha frequency band power as a function of condition and stimulus. Condition differentiation was not observed in the alpha frequency band for either stimuli (Fig. 2D,E, preferred stimulus: paired *t* test; t(14) = 0.958, *p* = 0.354; opponent stimulus: paired *t* test; t(14) = 0.404, *p* = 0.692), indicating again stimulus-specific MMN that was selectively reflected in both the raw ERP and specifically in the gamma frequency band.

**Figure 3:**
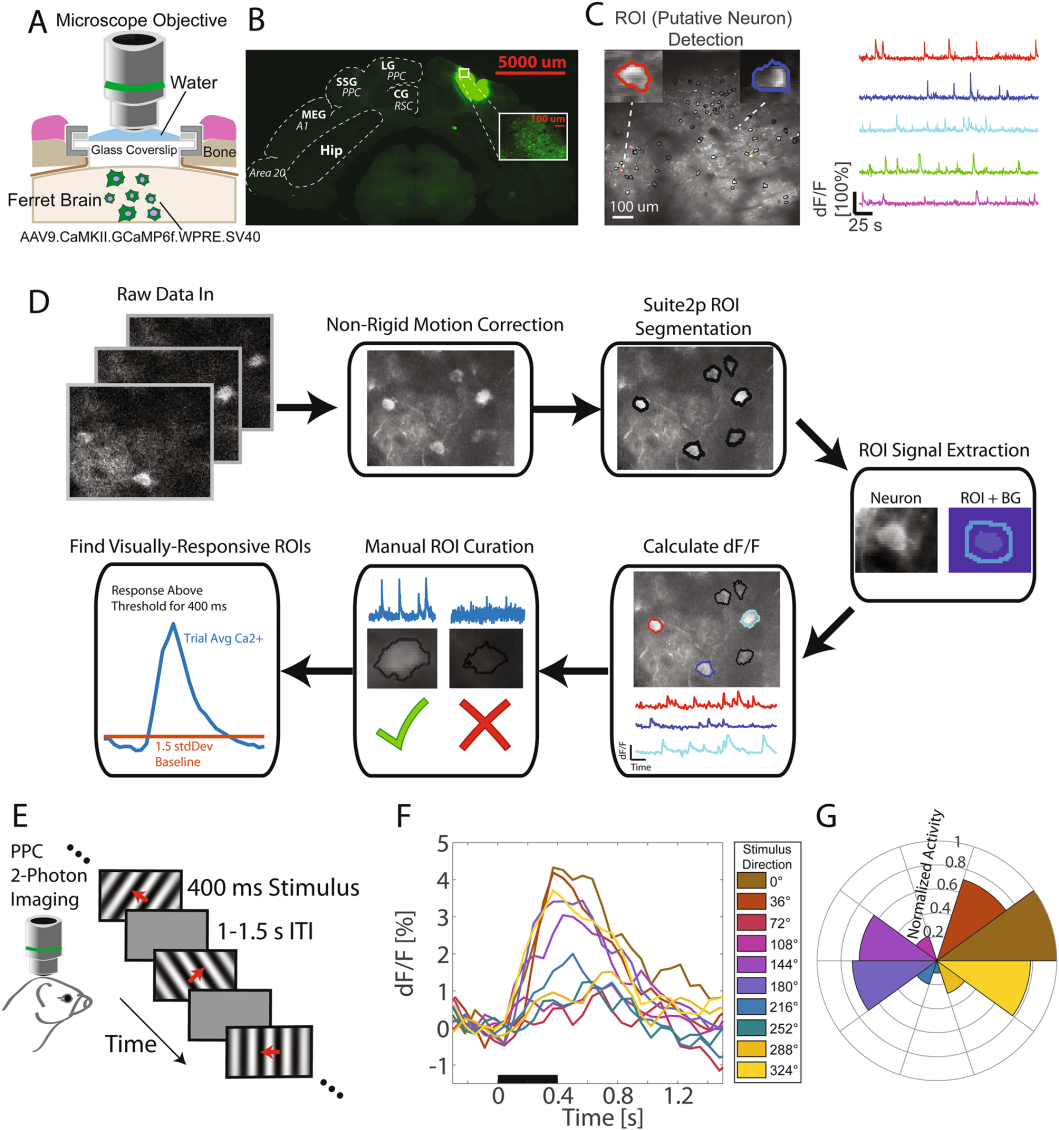
2-photon calcium imaging reveals stimulus-specific MMR in PPC. (**A**) Schematic of cranial window implant and calcium imaging setup. (**B**) Sample coronal section of PPC expression of GCaMP6f. Bold labels represent anatomical region names. Italicized labels represent functional region names. Red scale bar represents 5 mm distance. Inset: close up of recording area with cells labeled with GCaMP6f. Abbreviations: lateral gyrus (LG), posterior parietal cortex (PPC), cingulate gyrus (CG), retrosplenial cortex (RSC), suprasylvian gyrus (SSG), middle ectosylvian gyrus (MEG), hippocampus (Hip). Labels based on published ferret brain atlas^66^. (**C**) Left: Sample max fluorescence projection of a calcium imaging recording. Black contours represent regions of interest (ROIs) that were identified using a calcium imaging cell detection algorithm (Suite2p). Insets are close-ups of sample ROIs. Colored contours correspond to the sample fluorescence activity traces shown on the right. Signal extraction from ROIs was performed using a custom-designed algorithm with neuropil subtraction and dF/F calculation. (**D**) Calcium imaging preprocessing pipeline. Raw calcium imaging frames were first motion corrected then underwent ROI segmentation using the Suite2p toolbox^67^. ROI activity time-series were calculated by averaging across ROI pixels and subtracting the corresponding background (BG) activity defined by pixels in a ring around the ROI. Normalized activity was calculated as change in fluorescence over mean fluorescence across the session. To ensure all ROIs defined plausible cells, we manually curated the data based on ROI contours and time-series. Finally visually-responsive ROIs were chosen for subsequent analysis based on a stringent threshold for stimulus-evoked responses. (**E**) To assess direction tuning at the level of single neurons, head-fixed ferrets were presented a series of drifting gratings during 2-photon calcium imaging sessions. (**F**) Trial- and ROI-averaged event-related calcium responses for each drifting grating direction (in color) for a representative animal. Black bar represents the duration of stimulus presentation. (**G**) Polar histogram of normalized time-averaged (300–1,500 ms) calcium responses as a function of drifting grating direction. Note the two opposing directions that elicit the largest responses: the preferred and opponent stimulus.

With this finding that PPC electrophysiological signatures exhibit MMR as a function of stimulus preference, we next aimed to understand the single neuron basis of this phenomenon by using 2-photon calcium imaging in a separate group of animals (Fig. 3A–C; n = 4 ferrets). We isolated 73 putative neurons (or regions of interest—ROIs, Fig. 3D) that showed stringently-defined, robust visual responses to at least one condition and stimulus. This relatively low number of neurons for this passive visual stimulation paradigm matches with the general observation that activity in PPC is heavily task driven, as it is part of the dorsal fronto-parietal attention network^34^. During the initial 10-stimulus tuning assessment session (Fig. 3E), we found that average calcium responses from the local ROIs differed in magnitude as a function of drifting grating motion direction (Fig. 3F). Similar to Fig. 1, the calcium imaging results corroborated that PPC local tuning favored two opposing directions of motion where one direction elicited comparably stronger responses (preferred vs opponent stimulus; Fig. 3G, direction selectivity index = 0.3517).

**Figure 4:**
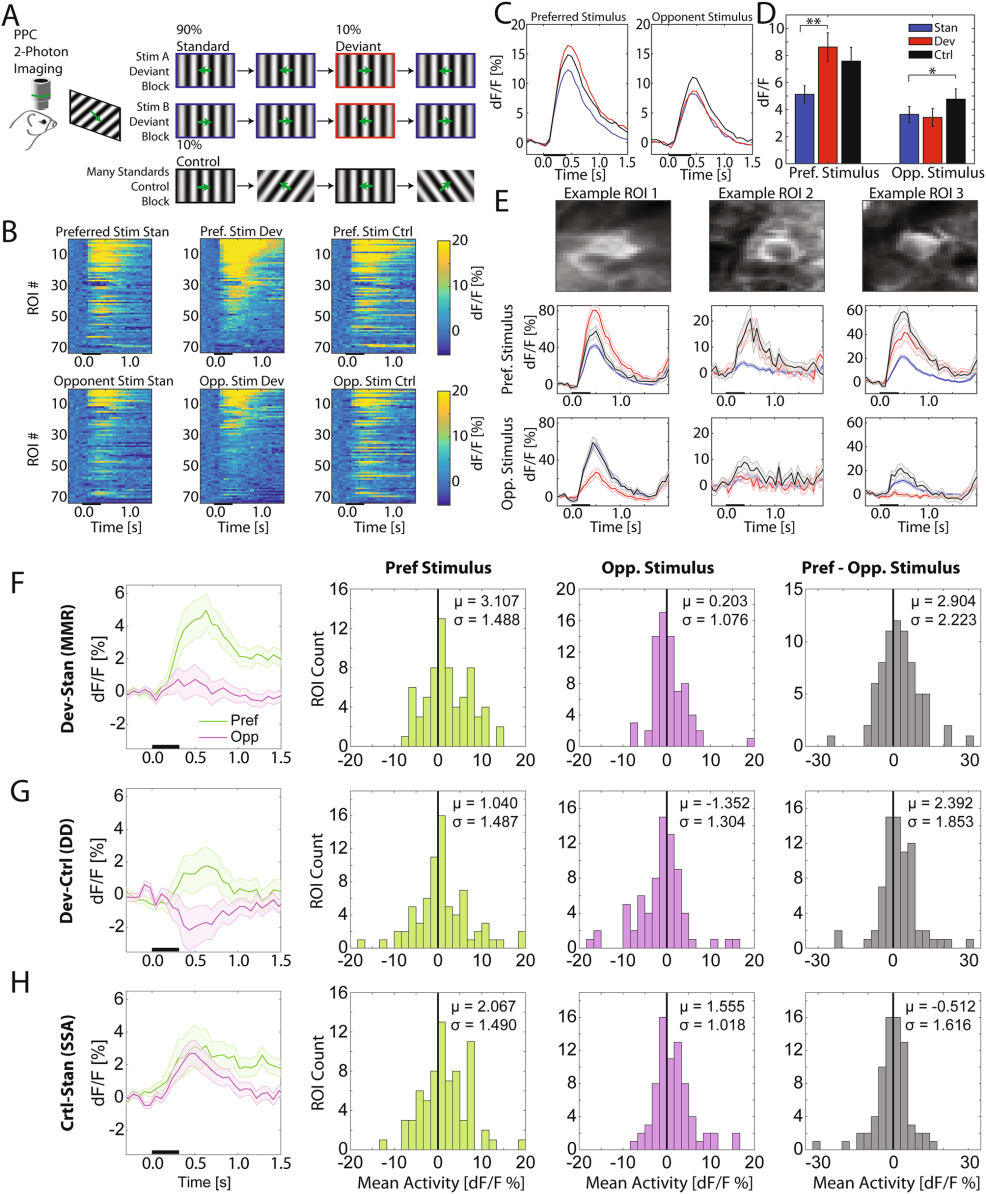
2-photon calcium imaging reveals stimulus-specific MMR is composed of stimulus-specific adaptation. (**A**) 2-photon calcium imaging experiment in PPC during visual oddball paradigm. Oddball paradigm is identical to the one utilized in the electrophysiology experiments (Fig. 1F). (**B**) Top: Heatmaps of each ROI’s trial-averaged activity as a function of time relative to stimulus onset for the preferred stimulus. ROIs are sorted by mean activity during the calcium response (300–1,500 ms) for the preferred stimulus in the deviant condition. The black bar on the x-axis represents stimulus presentation duration. Bottom: the same format but for the opponent stimulus. ROIs are resorted according to the opponent stimulus’ deviant condition activity. (**C**) ROI-averaged (n = 73) calcium responses for each condition (traces in color) and stimulus (left panel: preferred stimulus, right panel: opponent stimulus). The black bar on the x-axis represents stimulus presentation duration. (**D**) Time- and trial-averaged calcium responses as a function of condition and stimulus (n = 73 ROIs). *p < 0.01; **p < 0.001. Condition contrast definitions: mismatch response (MMR: deviant—standard), deviance detection (DD: deviant—control), stimulus-specific adaptation (SSA: control—standard). (**E**) Example ROIs (columns) mean fluorescence close-ups (top row), trial-averaged event-related calcium responses for the preferred stimulus (middle row), and responses for the opponent stimulus (bottom row). Note that ROI 1 shows both MMR (*p* < 0.001) and DD (*p* < 0.01) for the preferred stimulus. ROI 2 shows preferred stimulus MMR (*p* < 0.001), but no differences between conditions for the opponent stimulus (*p* > 0.05). ROI 3 shows MMR (*p* < 0.001) for the preferred stimulus. Error bars represent SEM. Statistical tests were unpaired *t*-tests with Holm-Bonferroni correction. (**F**) Left panel: Distribution of trial-averaged MMR across all ROIs for the preferred stimulus. Note the shift of the distribution towards higher values, indicating a significant MMR for the preferred stimulus. Middle panel: Distribution of trial-averaged MMR across all ROIs for the opponent stimulus. No MMR was found for the opponent stimulus. Right panel: Distribution of trial-averaged, stimulus-difference (preferred-opponent stimulus) MMR across all ROIs. Note the shift of the distribution towards higher values, indicating a difference in MMR between stimuli, specifically stronger MMR for the preferred stimulus. (**G**) Same format as in (**F**), but for DD. Note the significant shift of the opponent stimulus distribution towards negative values, indicating smaller responses in the deviant condition compared to the control. In the DD stimulus contrast, the distribution was shifted to higher values, indicating a significant difference between DD for the two stimuli. (**H**) Same format as in (**F**), but for SSA. Note that for both stimuli, the distributions were shifted to higher values, indicating significant SSA; however, SSA magnitude did not differ between stimuli indicated by the stimulus contrast.

Analyzing the condition-dependent responses for these visually-responsive ROIs using the subsequent oddball paradigm assays (Fig. 4A), we noticed select ROIs that displayed strong stimulus-evoked activity in the preferred stimulus deviant condition and comparatively weaker responses in the standard condition, indicating the presence of MMR (Fig. 4B). Indeed, ROI-averaged responses showed consistently higher activity for the preferred stimulus in the deviant condition compared to the standard condition across the period of 300–1,500 ms relative to stimulus onset (Fig. 4C,D, paired *t* test; t(72) = 3.71, *p* = 0.001; Holm-Bonferroni corrected). We did not find a significant difference between standard and deviant conditions for the opponent stimulus (paired *t* test; t(72) = 0.022, *p* = 0.982; Holm-Bonferroni corrected), further confirming that MMR occurs in parietal cortex in a stimulus-specific manner. On a neuron-by-neuron basis, we consistently found evidence for MMR for the preferred stimulus but not for the opponent stimulus (Fig. 4E).

The MMR can be dissected into two key components characterizing the surprise (deviance detection—DD) and adaptation (stimulus-specific adaptation—SSA) responses by comparing the control condition to the deviant or standard condition, respectively. To investigate the distribution of condition-dependent responses across ROIs, we reanalyzed the data by computing condition contrasts (ie. MMR, DD, and SSA) as a function of stimuli for each ROI (Fig. 4F,G,H). We computed MMR for each ROI in the analysis window (300–1,500 ms) and found that the mean of the distribution was shifted in the positive direction for the preferred stimulus but not the opponent stimulus (Fig. 4F 2nd column; preferred stimulus: μ = 3.107, σ = 1.488; opponent stimulus: μ = 0.203, σ = 1.076). Such stimulus-specific MMR was confirmed by computing for each ROI the difference between stimuli for the MMR (Fig. 4F, 4th column; μ = 2.904, σ = 2.223; paired *t* test; t(72) = 2.52, *p* = 0.011, Holm-Bonferroni corrected).

ROI-averaged responses the opponent stimuli in the standard condition adapted to a lower level compared to the control condition (paired *t* test; t(72) = 3.16, *p* = 0.007; Holm-Bonferroni corrected); a similar effect was observed for the preferred stimulus at trend level (paired *t* test; t(72) = 2.27, *p* = 0.052; Holm-Bonferroni corrected). In the distribution of time-averaged SSA across ROIs, both stimuli showed a similar amount of adaptation (Fig. 4H; preferred stimulus: μ = 2.067, σ = 1.490; opponent stimulus: μ = 1.555, σ = 1.018; stimulus contrast: μ = -0.512, σ = 1.616; paired *t* test; *t*(72) = 0.659, *p* = 0.512; Holm-Bonferroni corrected).

There was an absence of DD (deviant vs. control) for the preferred stimulus (paired *t* test; *t*(72) = 1.39, *p* = 0.168; Holm-Bonferroni corrected). Although not statistically significant, the deviant condition responses for the opponent stimulus were lower in magnitude than the control condition (paired *t* test; *t*(72) = 2.07, *p* = 0.085, Holm-Bonferroni corrected). In the time-averaged distribution, a negative DD indicated lower deviant activity compared to control (Fig. 4G 3rd column; μ = − 1.352, σ = 1.304). DD magnitude was significantly different between the two stimuli in the stimulus contrast (Fig. 4G, 4th column; μ = 2.392, σ = 1.853; paired *t* test; *t*(72) = 2.57, *p* = 0.011, Holm-Bonferroni corrected).

**Figure 5:**
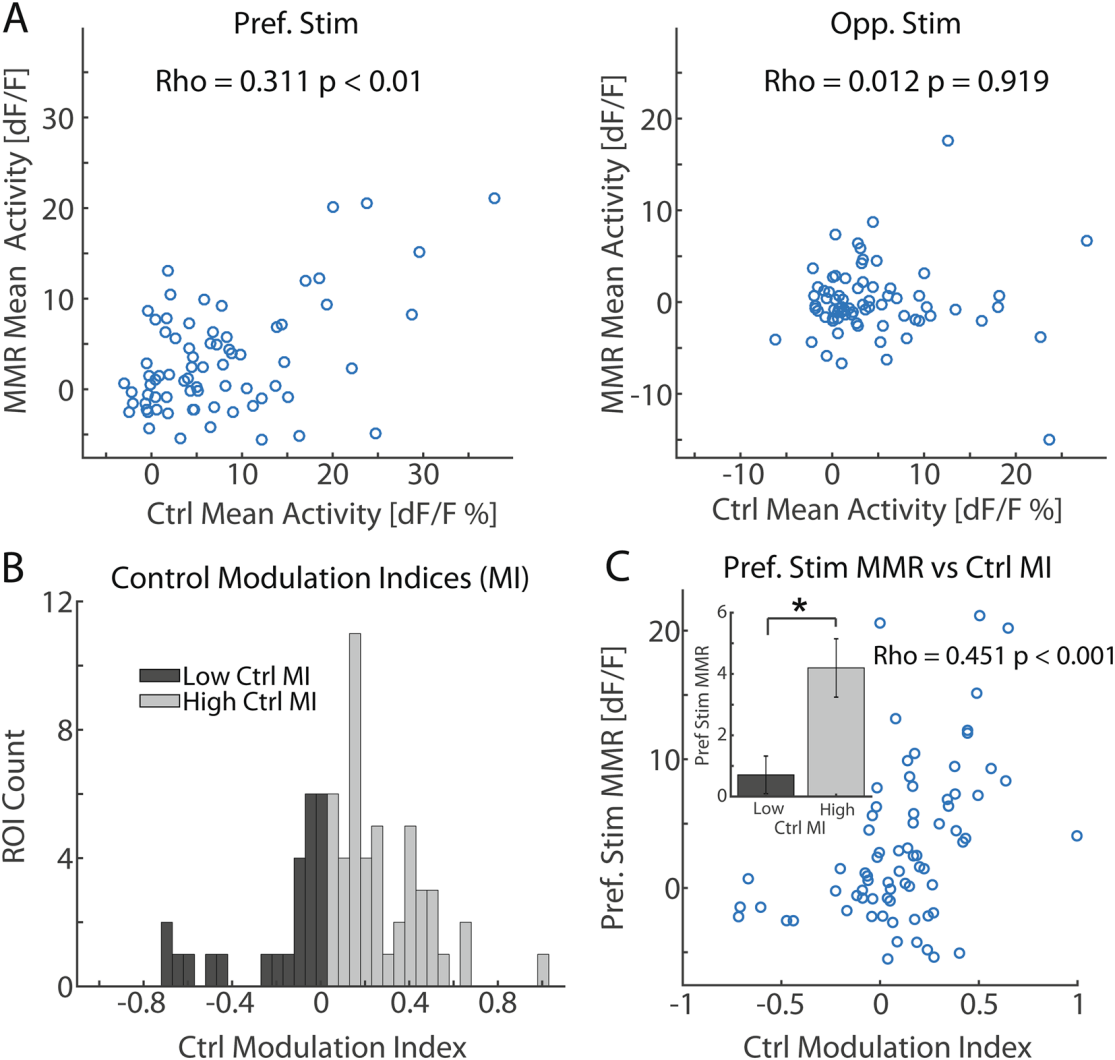
MMR scales with preferred stimulus control condition magnitude and stimulus-preference congruency with the population. (**A**) Left: Time-averaged (300–1,500 ms) MMR plotted against time-averaged control condition activity for each ROI for the preferred stimulus. A significant positive correlation (*p* = 0.008) between the two measurements indicates that ROIs with strong preferred stimulus control activity also show strong MMR. Right: same format as in the left panel but for the opponent stimulus. The opponent stimulus showed no clear relationship between MMR and control condition activity (*p* = 0.919). (**B**) Distribution of control condition modulation index (MI) across ROIs. A positive value represents congruent stimulus preference with the population average, whereas a negative value represents preference to the opponent stimulus. While there was variability in stimulus preference, the majority of ROIs showed congruent stimulus preference with the population. The distribution was split at 0 into high and low control MI groups. (**C**) Preferred stimulus time-averaged MMR plotted against the control condition MI for each ROI. A significant positive correlation between the two measurements indicate that ROIs with congruent stimulus preference with the population exhibit strong MMRs. These ROIs contributed strongly to the correlation (*p* = 0.00007) whereas ROIs with opponent stimulus preference seemed to show the same level of preferred stimulus MMR invariant of MI magnitude. Further, the high MI group exhibited higher magnitude preferred stimulus MMR compared to the low MI group (*p* = 0.020).

Taken together, 2-photon calcium imaging of PPC single neuron activity revealed the presence of stimulus-specific MMR, favoring the population-preferred stimulus; further, the main component of the MMR consisted of strong SSA. We next took advantage of 2-photon calcium imaging’s high spatial resolution by examining the relationship of MMR response to each ROI’s control condition response and stimulus preference. We found that the preferred stimulus MMR magnitude scaled with control condition response (Fig. 5A, left; Spearman’s correlation, Rho = 0.311, *p* = 0.008). In other words, ROIs with strong preferred stimulus control-condition activity showed strong MMR to the preferred stimulus. The opponent stimulus showed no clear relationship between MMR and control condition magnitude (Fig. 5B, right; Spearman’s correlation, Rho = 0.012, *p* = 0.919). These results suggest that the control condition response to the preferred stimulus, but not the opponent stimulus, predicts MMRs for individual neurons. However, these metrics do not show how MMR relates to stimulus tuning. Therefore, we calculated a modulation index (MI) from control condition responses where high and low values reflected preference congruent with the population or preference to the opponent stimulus, respectively. We noticed that neurons in the local field of view showed varying levels of stimulus preference where the majority of ROIs showed congruent stimulus preference to the population (MI values greater than 0) and a minority preferred the opponent stimulus (MI values less than 0) (Fig. 5B; population-congruent preference: n = 50; opponent stimulus preference: n = 23). MMR to the preferred stimulus showed a positive correlation with control condition MI (Fig. 5C; Spearman’s correlation, Rho = 0.451, *p* = 0.00007), such that ROIs with congruent population stimulus preference showed stronger MMR compared to ROIs with opponent stimulus preference (Fig. 5C; unpaired *t* test; *t*(72) = 2.38, *p* = 0.020). Together, these results agree with and provide understanding at the microscopic level of individual cells to the stimulus-specific nature of the MMR we found in the electrophysiology data.

## Discussion

Posterior parietal cortex is situated at the nexus between early visual and higher-order brain areas such as frontal cortex, and thus is ideally equipped to integrate basic tuning properties and environmental, contextual information. We investigated how direction preference in PPC influences processing of oddball conditions at the level of single neurons in the population using electrophysiology and 2-photon calcium imaging. We found that MMRs manifested in a stimulus-specific manner in both the single-neuron 2-photon calcium imaging and in the LFP activity: MMR occurred for the local population-preferred stimulus, but not for the opponent direction stimulus. Further, we found that both stimuli exhibited adaptation when presented in the standard, frequent conditions. Together, these results highlight single neuron-level computations that facilitate stimulus presentation frequency-dependent processing in a higher-order sensory area.

Animal models provide a unique window for viewing the cellular and circuit mechanisms underlying MMN; such spatial and temporal resolution is needed to understand what substrates are impaired in psychiatric disorders with sensory processing impairments and how to develop rationally-designed therapies to target specific regions or brain signatures involved in such deficits. Visual MMN has been observed in several animal models including non-human primates^14^, rats^15^, mice^12^, and rabbits^11^. It was recently found that both V1 and latero-intermediate area of the rat exhibits MMN in multi-unit firing responses to static images varying in texture^15^. An important visual oddball study in monkey inferior temporal (IT) cortex revealed stronger responses in the deviant condition compared to the standard (MMN), but failed to find a difference between deviant and many-standard equiprobable control condition^14^. Here we find similar results where the MMN can be explained primarily by adaptation; however, our single-cell 2-photon calcium imaging analysis revealed putative neurons that showed more prominent MMN and adaptation for one stimulus over the other. As a whole, such adaptation may reflect neuronal fatigue potentially due to firing rate adaptation, synaptic depression, or afferent control^35^. Indeed, recent reports suggest that the cellular mechanism responsible for repetition-related adaptation arises from afferent input (in particular, GABAergic) to adapted cells^36^.

Our results may provide insight to the mechanisms underlying the dysfunction of oddball-related sensory processing deficits in psychiatric disorders. One prominent mechanistic theory in schizophrenia research revolves around the role of N-methyl D-aspartate receptor (NMDAr)-dependent parvalbumin positive (PV+) interneuron activity^37–39^. Gamma oscillations arise from the interaction between excitatory pyramidal neurons and PV+ interneurons^40–42^. Further, NMDArs are causally involved in the regulation of PV+ neuron-mediated gamma oscillations^43^. As reduced gamma power has been associated with cognitive and sensory processing deficits in patients with schizophrenia^39,44^, it is hypothesized that NMDAr and PV+ interneuron dysfunction are underlying causes^37,45,46^. Indeed, NMDAr antagonist administration to humans^47–49^ and animal models^50–54^ recapitulates symptoms of schizophrenia such as psychosis, social withdrawal, cognitive-behavioral deficits, and relevant to this study, MMN presence. The results from our study are consistent with the role of gamma oscillations in oddball condition differentiation, and provide a further nuance that a higher-order sensory area, PPC, exhibits MMN in a stimulus-specific manner. Several works investigating the role of gamma oscillations in the directional routing of inter-regional information also supports the theory that MMN manifests as a feedforward signal in the gamma frequency band^33,55^. Alternatively, recent work has provided support for the causal involvement of somatostatin-positive (SOM+) neurons in MMN modulation^54^. Inspired by reports of inhibitory interneuron involvement in lateral inhibition and regulation of surround suppression^56,57^, one mechanistic explanation could be that these SOM+ neurons facilitating SSA by inhibiting pyramidal neurons during the standard condition^36^. Indeed, evidence shows that the suppression of activity may arise from SOM+ interneurons regulation of pyramidal neurons, as reversible inactivation of SOM+ neurons impair differentiation of stimulus presentation frequency in population activity^12^.

This study combines the respective strengths of 2-photon calcium imaging and electrophysiology, superior spatial and time resolution, to understand the mechanisms underlying the processing of oddball stimuli and conditions. Two-photon calcium imaging is capable of simultaneously visualizing the activity of 100’s of neurons, and allowed us to identify the handful of neurons relevant to oddball condition-dependent visual processing. This was particularly important for isolating sparsely distributed neurons across cortical space that showed oddball condition differentiation. Several models of MMN generation attempt to explain the single cell contribution to oddball condition differentiation^58^; however sparse activity in PPC make it difficult to record from numerous relevant neurons. Here, we show converging evidence of stimulus-specific MMN in both the electrophysiology and 2-photon calcium imaging data. It is also possible that using the GCaMP6f calcium indicator reduced our ability to resolve additional responsive neurons due to low signal-to-noise ratio (SNR); future studies could benefit from GCaMP6s which possesses superior fluorescence SNR. Nevertheless, the calcium imaging technology was crucial for novel dissection of how each individual neuron responded across conditions and how the MMR related to the tuning properties of each neuron.

The experimental paradigm used in this study was based on recent visual oddball studies for consistency and was used to accommodate previous reports of motion direction tuning in PPC^22,23^; however, we acknowledge limits in the insight that can be gained given this design. For example, our PPC tuning curves indicate strong orientation tuning. Since we focused on drifting stimuli that PPC was reported to respond strongly to, we are not able to examine how orientation tuning affects oddball condition differentiation. This experiment would be a natural extension for future work. Further, it may be construed that the lack of deviance detection in our results diminishes the significance of our study; however, by demonstrating that stimulus-dependent adaptation manifests across sparsely encoded cells, our findings build upon several seminal works that highlight the contribution of adaptation to MMN^16,17,59^. Our novel contribution to this adaptation hypothesis is that this attenuation of the ERP obligatory component may depend on single neuron stimulus preference. Finally, it is important to reconcile that we observed stimulus-specific MMR in the calcium imaging results, but SSA for both stimuli. We believe the stimulus-specific component of the MMR could arise from suppression of activity in the opponent stimulus’ deviant condition. Such a phenomenon could serve as a signal-to-noise amplification mechanism for stimulus presentation frequency differentiation in the local PPC circuitry. Although DD magnitude was not significant for the stimuli individually, the stimulus subtraction/contrast was significant; these findings may warrant further study of this phenomenon where deviant response amplitude is lower than that of the control condition.

Within the context of behavior, PPC has been extensively studied as a hub for processing salient and attention-orienting stimuli^60–62^. Given that the transition from a frequently presented stimulus to the rare stimulus can be surprising, one could interpret the stimulus-specific MMR as a differential signal that highlights the new, salient stimulus. Such a signal that represents salient stimuli could provide a foundation for goal-directed attention and stimulus target selection in behavior. The caveat of our study is that we can not truly measure saliency of the stimulus and condition to the animal without a behavioral measure; future studies would benefit from including an additional trial condition that requires the animal to make a response to rare or frequently-presented stimuli. PPC is an intermediate brain structure within the dorsal visual stream and serves as a hub for integrating visual information, prior stimulus history, and behavioral demands^61^. Visual cortex exhibits strong MMN to static images^12,15^; whereas PPC responds to moving/drifting stimuli^63^. It is possible that VC afferents holding MMN information are further processed by PPC to incorporate motion and stimulus-specific features. Such circuit-level transmission and integration of visual information across multiple visual cortices is understudied; the field would benefit from future studies utilizing targeted optogenetics to identify and stimulate VC presynaptic neurons that encode a particular orientation and observe MMN tuning changes in PPC. Finally, it is possible to explain the stimulus-specific MMN results in the context of the multiplexing of stimulus direction and orientation. Stimuli with opposing directions share the same orientation and therefore with the stimuli chosen in our study, PPC neurons may contend with both orientation and direction information. This is particularly important for the opponent stimulus where PPC activity could be dominated more by the stimulus orientation rather than direction. Since orientation remains invariant across standard and deviant conditions, this may explain the lack of MMN for the opponent stimulus.

Together, our results provide an extension to existing models of MMN whereby patches of PPC neurons up- and down-regulate activity as a function of stimulus and condition. For a given patch of PPC, the local neurons exhibit baseline direction tuning (the oddball control condition), where one direction elicits the strongest response (preferred stimulus) and the stimulus moving in the opposite direction shows comparable or lower magnitude responses. To differentiate the deviant and standard conditions, the local neurons that prefer the presented stimulus show substantial adaptation in the standard condition while activity during the deviant condition remains high; whereas the opponent stimulus does not show oddball condition discrimination in the neural activity. The topographic layout of PPC with respect to direction tuning (ie. spatially segregated patches of PPC exhibit preferred stimulus motion directions) is particularly important here^25,64,65^. The presence of a significant differential signal, the MMN, in the patch of PPC that prefers the presented deviant stimulus ultimately is relayed to downstream areas to affect behavior; whereas other spatial patches of PPC that prefer other stimulus directions do not show a MMN differential signal (that signals stimulus salience or importance). Such an organization may suggest specialization of stimulus presentation frequency differentiation across PPC spatial topography in a stimulus-specific manner. We recognize that follow-up work is required to substantiate this theory; particularly an exhaustive testing of static orientations and all motion directions in each permutation of standard, deviant, and control conditions across a larger area of PPC is required.

## Supplementary information

Supplementary Figures.

## Data Availability

Electrophysiological and 2-photon imaging data, as well as MATLAB code that was used to perform outlined analyses, can be made available from the corresponding author upon request.
