# Supplementary material for: Stimulus-specific regulation of visual oddball differentiation in posterior parietal cortex
Source: Sci Rep. 2020 Aug 18;10:13973. doi: 10.1038/s41598-020-70448-6 (PMC7435179; doi:10.1038/s41598-020-70448-6)
Supplement: Supplementary file 1 — Supplementary Figures. [file 41598_2020_70448_MOESM1_ESM.docx]

**Supplementary Materials for**

Stimulus-Specific Regulation of Visual Oddball Differentiation

in Posterior Parietal Cortex

Zhe Charles Zhou, Wei Angel Huang, Yiyi Yu, Ehsan Negahbani, Iain M Stitt, Morgan L Alexander, Jordan P Hamm, Hiroyuki K Kato, Flavio Fröhlich**^†^**

†Corresponding author. Email: Flavio_frohlich@med.unc.edu

**
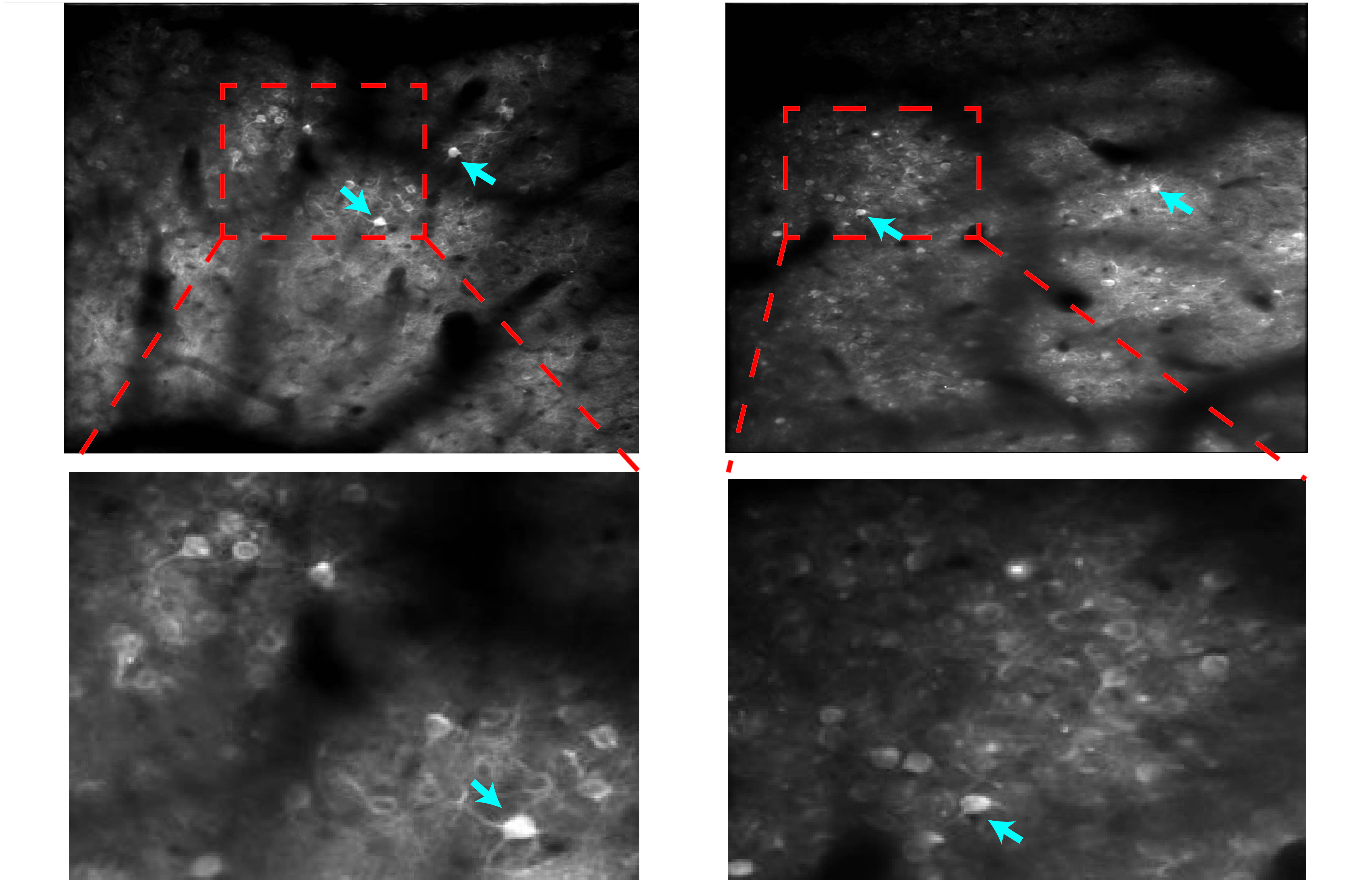
**

Supplementary Figure 1. Assessment of cells exhibiting overexpression of GCaMP6f

Top: Example mean fluorescence images of two animals expressing GCaMP6f. Blue arrows indicate cells that may be exhibiting nuclear filling. Bottom: close up of the regions outlined in red in the respective images above.


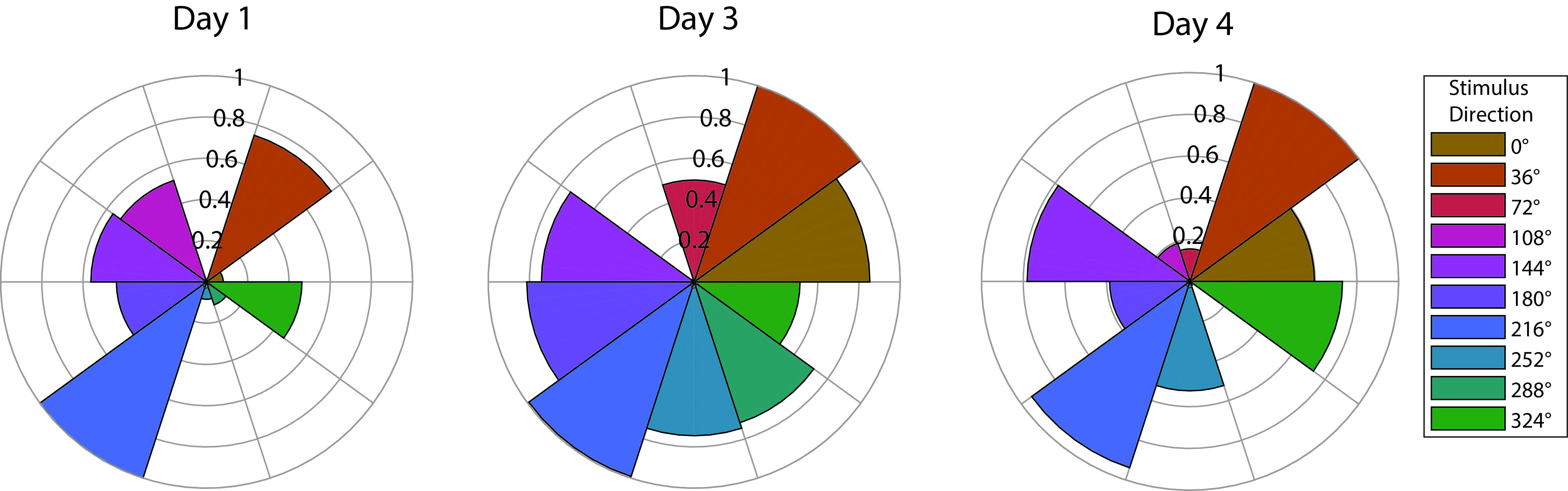


Supplementary Figure 2. Direction tuning for the animal shown in Fig. 1D across multiple recording days.


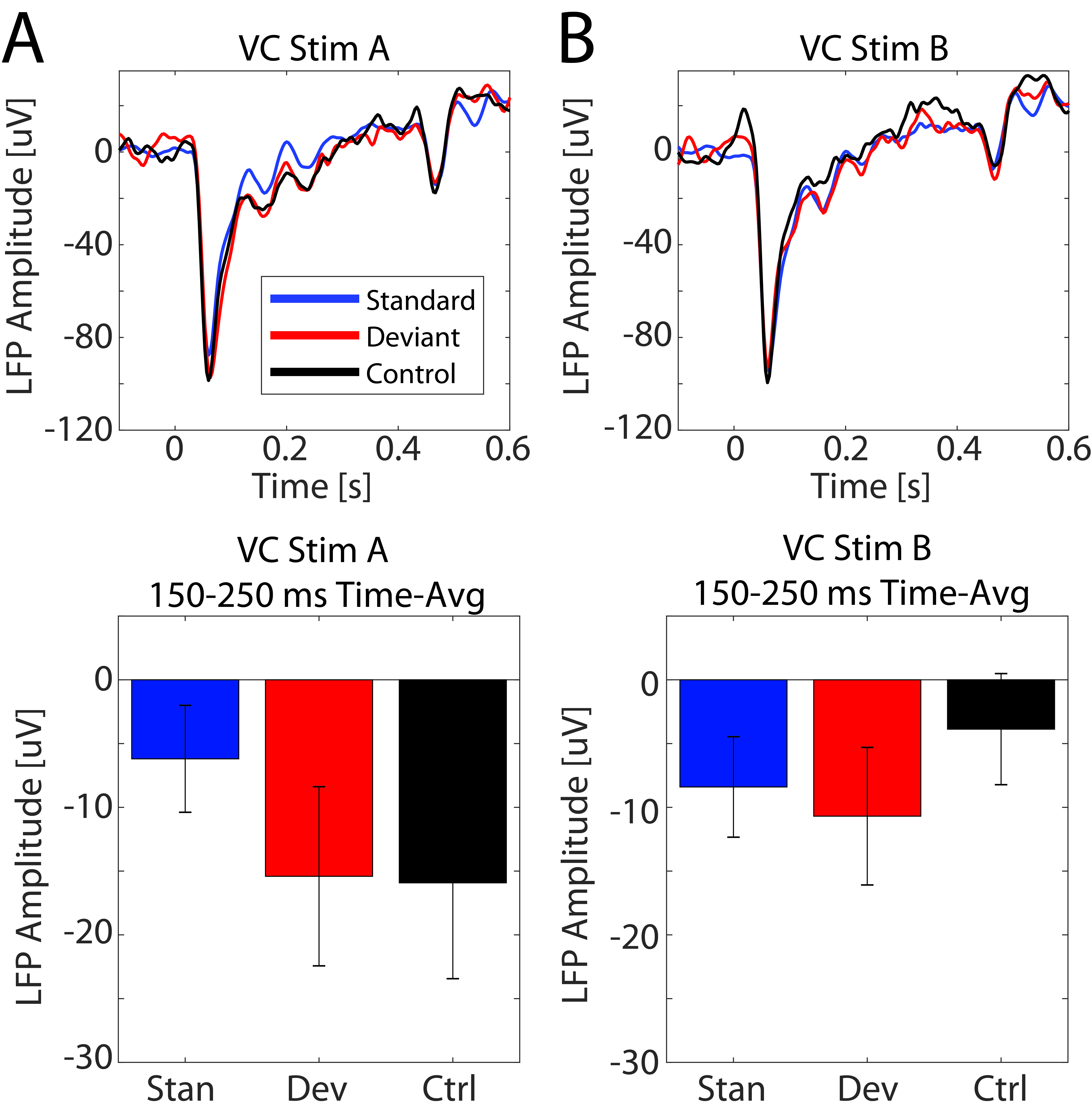


Supplementary Figure 3. Event-related potentials across oddball conditions in visual cortex

1. Top: Session-averaged (n = 7) event-related potentials from visual cortex (VC) across conditions for stimulus A (the preferred stimulus for PPC). Time is relative to stimulus onset. Bottom: Time-averaged ERP in the 150-250 ms time window relative to stimulus onset.
2. Top: Same as in A, but with stimulus B (opponent stimulus for PPC). Bottom: Same as in panel A but with stimulus B.


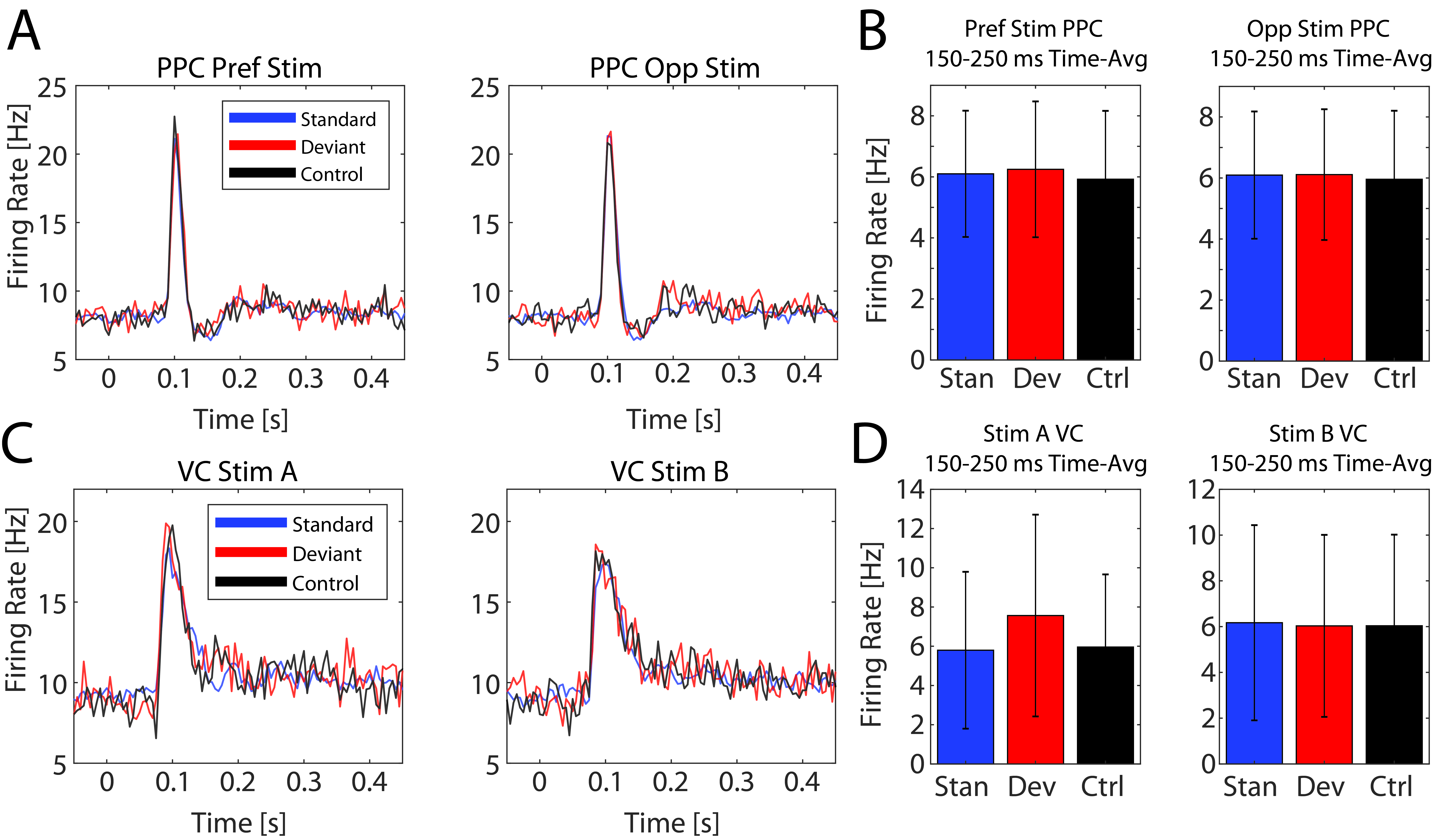


Supplementary Figure 4. Multi-unit firing rate activity across oddball conditions

1. Session-averaged (n = 15) firing rate (FR) peri-stimulus time histograms (PSTHs) from PPC across conditions for the preferred stimulus (left) and the opponent stimulus (right). Time is relative to stimulus onset.
2. Time-averaged (150-250 ms window) FR for each condition for the preferred stimulus (left) and the opponent stimulus (right).
3. Same as A but for visual cortex (VC) (n = 7).
4. Same as B but for VC.
